# Identification and Characterisation of Seed-Borne Fungal Pathogens Associated with Maize (*Zea mays* L.)

**DOI:** 10.1155/2021/6702856

**Published:** 2021-09-30

**Authors:** M. L. Goko, J. C. Murimwa, E. Gasura, J. T. Rugare, E. Ngadze

**Affiliations:** Department of Plant Production Sciences and Technologies, Faculty of Agriculture, Environment and Food Systems, University of Zimbabwe, P.O. Box MP167, Mount Pleasant, Harare, Zimbabwe

## Abstract

A research study was conducted to identify and characterise seed-borne fungal pathogens associated with maize (*Zea mays* L.) in storage. Seed-borne fungal pathogenic infections of maize were studied using seed samples collected from Gokwe South District in Zimbabwe. The agar plating method using PDA medium was used to detect fungal pathogens on the maize seeds. A total of 150 treatments were used for this experiment, which were replicated three times in a randomised complete block design (RCBD). Analysis of the grain showed the presence of *Fusarium moniliforme*, *Rhizopus stolonifer*, *Penicillium citrinum*, and mostly *Aspergillus* species, namely, *Aspergillus flavus*, *Aspergillus parasiticus*, *Aspergillus niger*, and *Aspergillus tamarii*. Significant differences (*p* < 0.05) between treatments were detected for the pathogens. A total of ten samples were used for mycotoxin determination, and all of them were 100% positive with aflatoxin total, zearalenone, fumonisin, and deoxynivalenol (DON) having an average of 0.255 ppb, 2.425 ppb, 2.65 ppb, and 0.07 ppb, respectively. The present study showed that most grain samples are contaminated with different species of fungi with mycotoxigenic potential. The data on the diversity and magnitude of pathogen infection by fungal species will have a significant effect even at the regional level for predicting the extent of pre- and postinfections. Measures to reduce mycotoxin contamination are needed for maize grains.

## 1. Introduction

Fungal pathogens cause contamination of grain crops including maize (*Zea mays* L.) prior to harvesting or after harvesting. This contamination gives rise to several fungal seed-borne pathogens that can be identified on seeds or cause a number of diseases [1]. Certain seed-borne fungal pathogens produce mycotoxins, which are substances that cause the deterioration of grain quality, poor germination capacity, and reduced vigour [2]. Mycotoxins are secondary metabolites that degrade a variety of substrates mostly composed of carbon such as plants. Fungal pathogens that produce mycotoxins that were detected on maize include *Aspergillus*, *Fusarium*, *Penicillium*, and *Rhizopus* species. Hussain et al. [3] reported a number of mycotoxins such as aflatoxins and sterigmatocystin, which are produced by *Aspergillus* species. *Fusarium* species produce a broad spectrum of mycotoxins that are of notable importance, such as trichothecenes of A and B types. Deoxynivalenol (DON) and nevalenol are the most important B-type mycotoxins. The other mycotoxins also produced by this genus are fumonisins, zearalenones, moniliformins, fusaproliferins, diacetoxyscirpenol, beauvericin, and fusarenone [4]. Reference [5] reported that *Penicillium* species also produce mycotoxins known as vomitoxins and zearalenones. Based on the research done by Jennessen et al. [6], *Rhizopus* species were reported to produce mycotoxins known as rhizonins.

According to [7], mycotoxins cause a reduction in the quality of the harvested crops and cause health problems in humans and animals. Mycotoxins vary in their structures, hence resulting in a great variation of their effects [4]. Seed-borne fungal pathogens have been found to affect the growth and productivity of crop plants. They result in seed necrosis, seed abortion, rotting of seed, reduction, or elimination of germination capacity as well as seedling damage resulting in the development of disease at later stages of plant growth by systemic or local infection [3]. The resulting phytotoxic characteristics are dose-related and differ from one mycotoxin to another. Mycotoxins also pose economic impacts including loss of human as well as animal life. They increase health and veterinary care costs due to investments in research and applications to reduce their problems. Moreover, aflatoxins cause losses in livestock and poultry production from aflatoxin-contaminated feeds that cause death, immune system suppression, reduced rates of growth, and reduction in feed efficiency. Hydroxylated derivatives of aflatoxins are formed by lactating animals and excreted in milk after mycotoxin consumption. This makes contaminated milk unsafe for human consumption.

Identification and characterisation of seed-borne fungal pathogens associated with maize are important in ensuring effective control of pathogens that produce mycotoxins [8]. Atanda et al. [9] reported that fungal pathogens are ubiquitous; therefore, there is a need to avoid improper storage as well as poor agronomic practices leading to mycotoxin production. The characterisation also helps note the appropriate choice of variety, harvest and storage, moisture, and aeration considerations to limit mycotoxin biosynthesis. This is important because small numbers can rapidly multiply under favourable conditions causing contamination [10]. The fungal seed-borne pathogens thrive in various conditions. Mycotoxin production during pre- or postharvest handling of agricultural commodities depends on several intrinsic factors such as moisture content, water activity, substrate type, plant type, and nutrient composition [11]. Extrinsic factors such as climate, temperature, and oxygen level also play a role together with processing factors such as drying, blending, adding preservatives, and handling of grains. Moreover, implicit factors such as insect interactions, fungal strain and its specificity, and microbiological ecosystem are also involved. Atanda et al. [9] showed that the growth of fungi in storage depends on the composition of nutrients in the grain, moisture, and temperature conditions as well as biotic factors such as competition or the presence of stored product insects.

Hussain et al. [3] reported a number of mycotoxins produced by *Aspergillus* species such as aflatoxins and sterigmatocystin. *Aspergillus flavus* causes Aspergillus ear roots, which is a serious problem when infected ears are stored at high moisture content. A number of these *Aspergillus* species can infect maize prior to harvesting. *Aspergillus niger* is the most common, and it produces powder-like black masses of spores that surround the kernels and the cob. In contrast, *Aspergillus glaucus*, *A*. *flavus*, and *Aspergillus ochraceus* normally form yellow-green masses of spores. On the other hand, *Aspergillus parasiticus* is ivy green and less common in maize. *A*. *flavus* and *A*. *parasiticus* produce mycotoxins known as aflatoxins that are harmful to animals as well as to human beings [12].

Reference [5] stated that mycotoxins produced by *Penicillium* species result in Penicillium ear roots. *Penicillium oxalicum* is known to cause damage, and there are other species that may also be pathogenic producing mycotoxins. In a number of cases, infection by such fungal pathogens will be a result of ear damage caused by insects. A powder that appears light blue-green develops between the kernels and on the cob surface. Those kernels with fungal colonies normally become bleached and streaked [13].

*Fusarium* species produce a number of mycotoxins that are of notable importance. Deoxynivalenol (DON) as well nevalenol are the most important B types produced by *Fusarium* species. Other mycotoxins common to this genus are fumonisins, zearalenones, moniliformins, fusaproliferins, diacetoxyscirpenol, beauvericin, and fusarenone [4]. *Fusarium* cob rot or ear rot is a result of *Fusarium* species. It is a seed-borne infection caused by many *Fusarium* species such as *Fusarium verticillioides*. The development of *Fusarium* cob rot is caused by warm, dry weather prior to harvesting, for example, at or after flowering. Danielsen et al. [14] regarded *F*. *verticillioides* as the major producer of the mycotoxin fumonisin. A number of kernels can be affected on the whole cob. The infection will be noticed by the formation of a fungal growth that is whitish pink-lavender. This usually appears at the tip of the ear and is mostly facilitated by ear damage. *Fusarium* spp overwinter on infested maize stalks, and the asexual spores maybe dispersed to the unifected plants by wind or irrigation water. During the growing season, spores may infect the silks, which are formed during flowering as they move in the air or are systemically transferred. Management of insects and good storage practices reduce the risk of mycotoxin contamination [15]. The use of resistant hybrids can also help reduce pathogen infection.

*Fusarium graminearum* and *F. verticillioides* species cause cob rot, ear rot, or pink ear rot. Gibberella cob rot is favoured by cool, moist conditions before harvesting, especially at flowering. It is therefore more prominent in wetter, cooler growing regions [13]. There are several mycotoxins that are produced *by F*. *graminearum*, such as zearalenone and trichothecene mycotoxin groups. The disease is mainly caused by monoculture cropping of maize, rotation of maize winter cereal as well as plant stress at the stage of grain filling. An infected plant shows the development of fungal growth that is reddish pink or whitish pink at the tip of the cob. The husks tend to bind to the kernels with or without some black fruiting bodies on the outer leaves. Contamination is brought about by airborne spores. The use of appropriate agronomic practices, resistant hybrids, prompt harvesting, and proper storage can minimise the risk.

The practice of retaining seeds and poor certification procedures provide avenues for infection of seeds by a number of diseases [12]. Seeds carrying such pathogens are detrimental to the production of crops because they reduce seed viability and seedling vigour. This would result in a decrease in the population of established seedlings and hence reduced yield. Residues or seeds can act as sources of inoculums resulting in further infection. Chemical control cannot fully suppress fungi on the seeds, so further disease development may result [16]. The pathogens may completely result in the deformation of seeds and the concomitant erosion of grain quality [2]. Therefore, seed-borne fungal pathogen identification and characterisation are important components of integrated disease management in order to reduce contamination of grain with mycotoxins. Although chemical control can be effective to some extent, full reliance on it has not been fully advised, but rather cultural and biological control methods [17]. Above all, suitable agronomic management practices have to be done, and suitable storage facilities are needed to limit contamination, development, and growth of such seed-borne pathogens [18].

## 2. Materials and Methods

### 2.1. Study Area

The experimental work for the isolation and identification of seed-borne fungal pathogens of maize grain samples obtained from Gokwe South District was carried out at the University of Zimbabwe Pathology laboratory in the Department of Plant Production Sciences and Technologies.

### 2.2. Experimental Design

One hundred and fifty treatments used in this experiment were replicated three times in a randomised complete block design (RCBD). The blocks were three benches on which treatments (Petri dishes with ten seeds from one storage facility per treatment) were placed for incubation at room temperature at 25 °C for seven days.

### 2.3. Isolation Using the Agar Plating Method

Infected maize grains were surface sterilised in sodium hypochlorite (NaClO_2_) for three minutes [3]. Thereafter, the seeds were rinsed three times using sterile distilled water and dried on a sterile blotter paper for two minutes. Potato dextrose agar (PDA) was used in the fungal isolation procedure [19]. A maximum number of ten seeds were plated on the sterile PDA poured into each Petri dish. The ten seeds placed in each Petri dish had three replicates and were incubated at 25°C at room temperature. The seeds were arranged uniformly making sure that they were equidistant from each other. Subculturing was done using PDA to obtain pure cultures. All these procedures were done under sterile working environments.

### 2.4. Identification Using Microscopy

The process of identifying fungal seed-borne pathogens that formed an overgrowth on maize grains was done using a compound microscope (model AusJena Laboval 4 and Leits Laboraux K) as described by Krnjaja et al. [20]. Visual assessment of the presence and characteristics of the fruiting structures was done using spore colour and colonisation. Resolution of light appearing structures was aided by placing the Petri dishes on a black surface.

The isolated fungi fruiting structures were examined after slide preparation. The seed-borne fungal pathogens were also identified through the use of taxonomic features such as conidia and hyphae [3]. This was made possible through the use of identification manuals and slides that were preserved and kept in the Plant Pathology laboratory.

### 2.5. Mycotoxin Detection

Samples showing high infection for each pathogen were selected for the enzyme-linked immunosorbent assay (ELISA) test. These are shown in Table 1.

The mycotoxin identification procedure was done according to the method described by Krnjaja et al. [21], with a few modifications of the procedures involved. The ELISA method was used in the detection of aflatoxin, total deoxynivalenol (DON), fumonisins, and zearalenone in a total of ten samples. Samples were ground using an analytical mill (IKA A11, Germany), and the powder was kept in a refrigerator at 4°C awaiting analyses. One gram of NaCl was mixed with 5 g of each sample and then homogenised in 25 ml of 70% methanol. The mixture was placed in a 250 ml Erlenmeyer flask, and shaking was done manually. Following this procedure, the homogenate was filtered using Whatman filter paper no. 1. Analysis of filtrate was done using ELISA kits (R-Biopharm AG). Absorbance was measured at a wavelength of 450 nm on an ELISA reader model (Biotek EL × 800 TM, USA). The procedure involved the insertion of a sufficient number of microtiter wells into the microtiter holder for all standards, and the samples were run in duplicate. The standard and sample positions were recorded. Fifty microlitres of standard or prepared samples to separate wells were pipetted using a new pipette tip for each standard sample. Fifty microlitres of enzyme conjugate were added to the bottom of each well. Thereafter, fifty microlitres of anti-deoxynivalenol, anti-fumonisin, anti-aflatoxin total, or anti-zearalenone antibodies were added as a solution to each well separately. Mixing by shaking the plate manually was done gently and incubated for 30 minutes at room temperature (20–25°C/68–77°C).

The liquid was removed from the wells and placed on an absorbent paper for the complete removal of the liquid contained in the wells. A total of 250 *μ*L washing buffer was added, and the liquid was poured out again from the wells. The washing procedure was repeated two times. One hundred microlitres of substrate/chromagen were added to each well. Mixing was then done gently by shaking the plate manually and incubating for 15 minutes at room temperature (20–25°C) in the dark. One hundred ul of stop solution was added to each well. Mixing was done by gently shaking the plate manually, and the absorbance was measured at 450 nm. This was read within 30 minutes after the addition of the stop solution.

### 2.6. Data Collection

Counts of seeds infected with fungal pathogens were recorded based on the type of fungal growth on the seeds. The fungal pathogens were distinguished through a visual assessment, where the colours of the colonies were identified by naked eyes. Microscopy was used in the conformation of the fungal genera identified by visual assessment. This showed the disease incidence of seed-borne fungal pathogens for each sample. Furthermore, the ELISA method was used in the detection of the mycotoxins, aflatoxin total deoxynivalenol (DON), fumonisin, and zearalenone (ZON) in a total of ten samples.

### 2.7. Data analysis

Fungal pathogen count data were subjected to analysis of variance (ANOVA), and the means were obtained using Minitab 16. Further analysis was done using GenStat 14^th^ edition and nonparametric tests using Minitab 16^th^ edition. All significant mean differences were separated using Tukey's test at a 5% significant level. The quantities of mycotoxins were determined using the values calculated for each particular mycotoxin standard entered in a system of semilogarithmic graph paper with the concentration in ppb. The concentration of each mycotoxin was then read from the calibration curve.

## 3. Results

### 3.1. Identification of Seed-Borne Fungal Pathogens from Different Storage Facilities

Storage facility had a significant (*p* <0.05) effect on the mean seed infection. Containers recorded the highest *Fusarium moniliforme* infection, while kitchen recorded the lowest (Table 2). The highest *Rhizopus stolonifer* infection was noted in cotton bags, and none was detected in the container. The highest *Penicillium citrinum* infection was recorded in house sacks, and the container recorded no infection (Table 2).

Traditional granary and house sacks recorded significantly (*p* < 0.05) the highest levels of *A*. *flavus* infection, and none was detected in the container. A significantly (*p* < 0.05) high level of *A*. *parasiticus* infection was noted in the kitchen as there was no significant difference in the amount of infection recorded in the other environments (Table 3). Seeds stored in the traditional granary recorded significantly (*p* < 0.05) the highest level of *A*. *niger* infection (Table 3).

**Figure 1:**
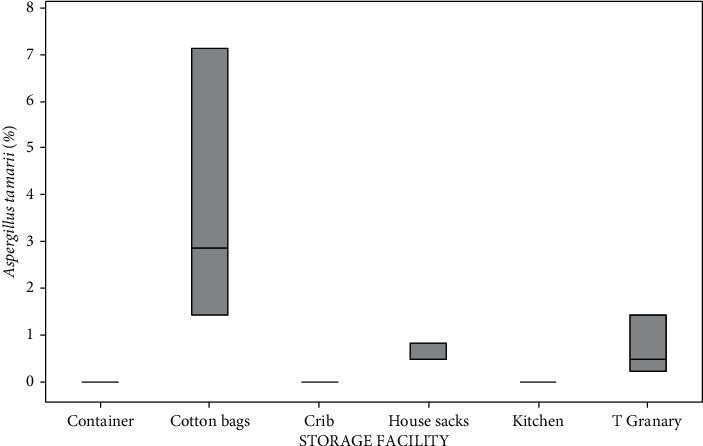
Boxplot of *Aspergillus tamarii* detection in different storage facilities.

Storage facility had a significant (*p* < 0.05) effect on the mean seed infection. Data from Figure 1 show that cotton bags recorded the highest *A*. *tamarii*, while container crib and kitchen recorded the lowest level of infection. House sacks and traditional granary represented the intermediate infection (Figure 1).

### 3.2. Identification of Seed-Borne Fungal Pathogens in Different Stored Maize Varieties

**Figure 2:**
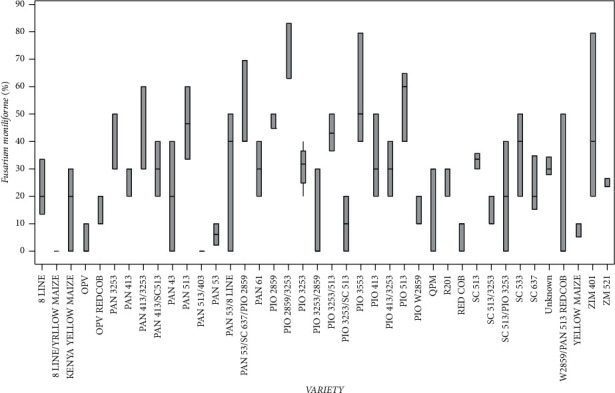
Percentage of mean seed infection for each variety of *Fusarium moniliforme*.

Variety type had statistically significant (*p* < 0.05) differences in *F*. *moniliforme* infection. The eight line/yellow maize varieties recorded the highest *F*. *moniliforme* infection while *Pan* 513/403 recorded the lowest (Figure 2). The other varieties showed significantly (*p* < 0.05) intermediate infection of *F*. *moniliforme*.

**Figure 3:**
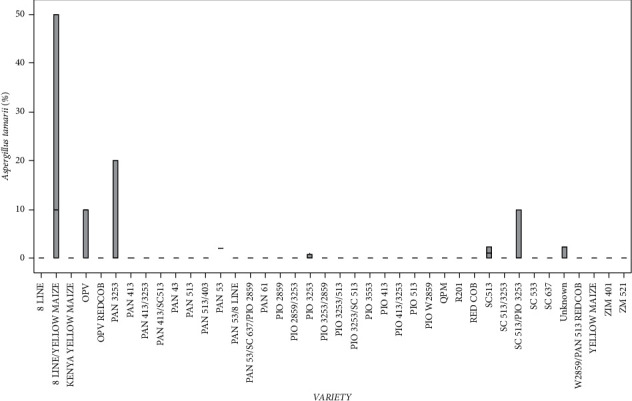
Percentage of the mean seed infection for each variety of *A*. *tamarii*.

There were statistically significant differences (*p* < 0.05) in *A*. *tamarii* infection between the varieties. The eight line/yellow maize had the highest infection of *A*. *tamarii* (Figure 3). The eight LINE, Kenya YELLOW MAIZE, OPV RED COB, PAN 413, PAN 413/3253, PAN 413/SC513 PAN 43, PAN 513/403, PAN 513, PAN 53/8 LINE, PAN 61, PAN 53/SC 637/PIO2859, PIO 2859, PIO 2859/3253, PIO 3253/2859, PIO 3253/513, PIO 3253/SC 513, PIO 3553, PIO 413, PIO 413/3253, PIO 513, PIO W2859, QPM, RED COB, R201, SC 513/3253, SC 533, SC 637, W2859/PAN 513, REDCOB, YELLOW MAIZE, ZIM 401, and ZM 521 varieties had the lowest *A*. *tamarii* infection, and none was detected in the other varieties (Figure 3).

**Figure 4:**
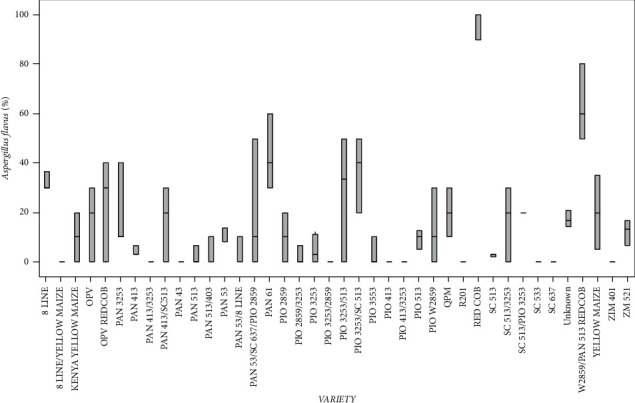
Percentage of mean seed infection for each variety of *A*. *flavus*.

Variety type had a significant (*p* < 0.05) effect on pathogen infection. RED COB recorded the highest *A*. *flavus*, whereas 8 LINE/YELLOW MAIZE, PAN 413/3253, PAN 43, PIO 3253/2859, PIO 413, PIO 413/3253, R201 SC 533, SC 637, and ZIM varieties recorded the lowest (Figure 4).

**Figure 5:**
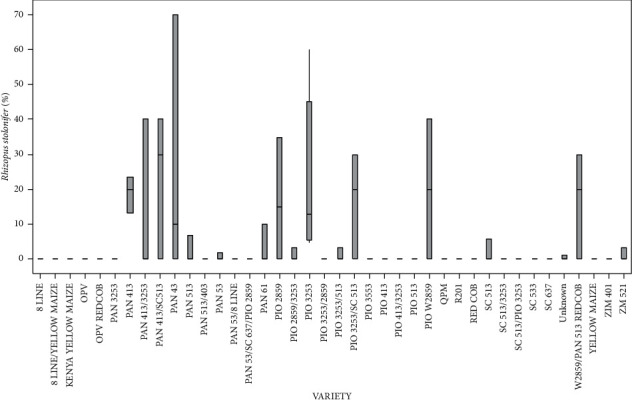
Percentage of the mean seed infection for each variety of *R*. *stolonifer*.

Variety type had a significant (*p* < 0.05) effect on the level of *R*. *stolonifer* infection. *Pan* 413 showed the highest *R*. *stolonifer* infection (Figure 5). PIO 3253 recorded the average pathogen infection, while none was detected in the other varieties.

**Figure 6:**
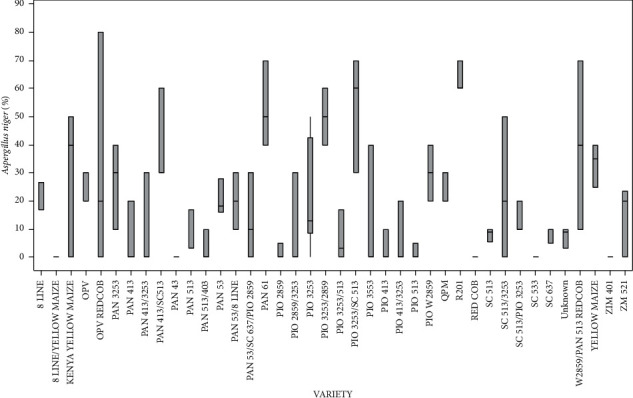
Percentage of the mean seed infection for each variety of *Aspergillus niger*.

The level of infection for the various varieties of the seed-borne fungal pathogen *A*. *tamarii* showed statistically significant differences (*p* < 0.05). The varieties OPV/REDCOB recorded the highest *A*. *niger* infection, while 8 LINE/YELLOW MAIZE, PAN 43, RED COB, SC 533, and ZIM 401 recorded the lowest (Figure 6).

**Figure 7:**
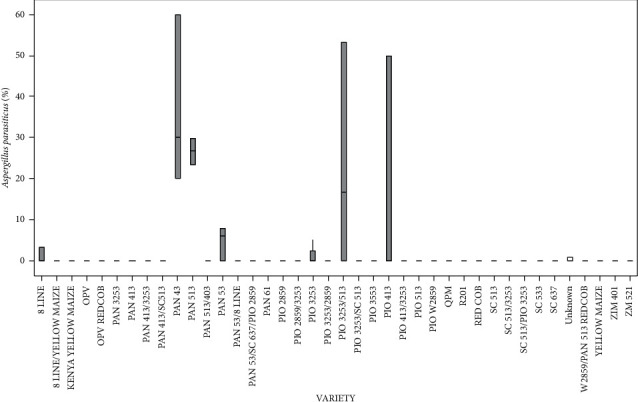
Percentage of the mean seed infection for each variety of *A*. *parasiticus*.

Variety type had a significant (*p* < 0.05) effect on *A*. *parasiticus* infection (Figure 7). PAN 43 recorded the highest pathogen infection. 8 LINE/YELLOW MAIZE, Kenya YELLOW MAIZE, OPV, OPV/REDCOB, PAN 3253, PAN 413, PAN 413/3253, PAN 413/SC513, PAN 513/403, PAN 53/8 LINE, PAN 53/SC 637/PIO2859, PAN 61, PIO 2859, PIO 2859/3253, PIO 3253/2859, PIO 3253/SC 513, PIO 3553, PIO 413/3253, PIO 513, PIO W2859, QPM, R201, RED COB, SC 513, SC 513/3253, SC 513/PIO 3253, SC 533, SC 637, W2859/PAN 513 REDCOB, YELLOW MAIZE, ZIM 401, and ZM 521 varieties showed the lowest pathogen infection (Figure 7).

**Figure 8:**
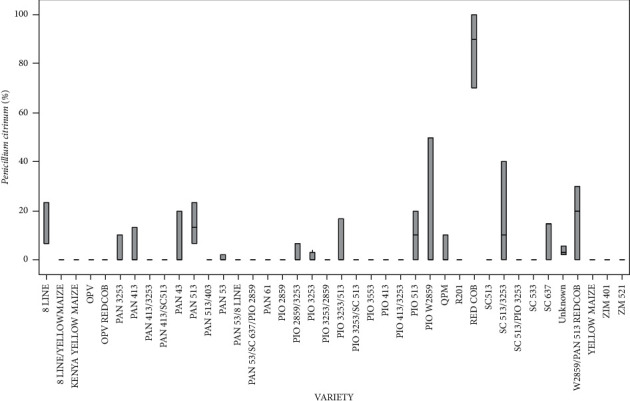
Percentage of the mean seed infection for each variety of *P*. *citrinum*.

The level of infection for the various varieties of the seed-borne fungal pathogen *P*. *citrinum* had statistically significant differences (*p* < 0.05). RED COB recorded the highest *P*. *citrinum* (Figure 8). 8 LINE/YELLOW MAIZE, Kenya YELLOW MAIZE, OPV REDCOB, PAN 413/3253, PAN 413/SC513, PAN 513/403, PAN 53/8 LINE, PAN 53/SC 637/PIO2859, PAN 61, PIO 2859, PIO 3253/2859, PIO 3253/SC 513, PIO 3553, PIO 413, PIO 413/3253, SC 513, SC 513/PIO 3253, SC 533, YELLOW MAIZE, ZIM 401, and ZM 52 varieties recorded the lowest pathogen infection (Figure 8).

### 3.3. Mycotoxin Identification

A total of 10 samples selected as representative among the samples were 100% positive with aflatoxin total, zearelenone, deoxynivalenol (DON), and fumonisin with an average of 0.255 ppb, 2.425 ppb, 2.65 ppb, and 0.07 ppb, respectively (Table 4).

## 4. Discussion

The study was designed to identify and characterise seed-borne fungal genera present in stored maize grain and quantify the amount of mycotoxins, which they produce. The isolation and identification procedures using the agar plating method and microscopy revealed a diverse nature of fungal seed-borne pathogens in maize samples collected from Gokwe South. The fungal pathogenic isolates, namely, *F*. *moniliforme*, *A*. *tamarii*, *A*. *parasiticus*, *A*. *niger*, *A*. *flavus*, *R*. *stolonifer*, and *P*. *citrinum* were identified. Four types of mycotoxins, namely, fumonisins, zearalenone (ZON), deoxynivalenol (DON), and aflatoxin total were detected in the maize samples collected from smallholder farmers in Gokwe South District. *F. moniliforme*, *A*. *tamarii*, *A*. *parasiticus*, *A*. *niger*, *A*. *flavus*, *R*. *stolonifer*, and *P*. *citrinum* were detected in maize grain stored in cotton bags, kitchen, traditional granaries, house sacks, crib, and containers. Research findings of this study depict that pathogenic fungal isolates occurred in diverse infection incidence among different storage facilities. The possible causes for such results are abiotic factors, which can directly affect the relative frequency of fungal populations in stored grain [22]. Moreover, there were significant differences in pathogen infection in relation to the storage facilities for all species identified. The results for the isolation and identification of seed-borne fungal pathogens associated with stored maize grain were in agreement with the findings of Krinjaja et al. [21] and Amadi and Adeniyi [10].

Our findings show that *Aspergillus* species were the most predominant species in stored maize grain. These findings concur with the literature that *Aspergillus* moulds can be attributed to factors such as warmth and high relative humidity with low temperatures, which may result in improper drying of the maize as well as high temperatures with drier conditions, which predispose maize to moulds in the field or in storage [23]. Srivastava et al. [13] also reported that there are weather conditions, which favour the fungal establishment in maize, hence threatening its safety during storage as well. Studies done by [24] also support the findings from this study that seed-borne fungal pathogens can infect maize preharvest and increase mycotoxin levels under different storage facilities if conditions are poorly managed. Some of the other favourable conditions resulting in infection involve stress due to drought, poor nutrition of plants, plant diseases, plant pests, weeds, and high plant populations. Reference [25] showed that varying conditions such as water and temperature in various storage facilities determine the fungal growth. However, the differing conditions in each storage facility facilitate the growth of particular fungal pathogen as they differ in their requirements for development as well as mycotoxin production.

Variety influenced the level and type of infection detected on the seed as different pathogens were detected on different varieties. A number of factors contribute to the differential pathogen infection of different varieties. The presence of storage insects in the grain could also be regarded as a contributing factor to the level and type of infection detected as some varieties are more susceptible to insect damage than others. However, studies done by Hell [26] indicated that husk cover plays a role in the protection of kernels against insect pests, which create openings for pathogen entry. According to the study done by Cardwell et al. [27], varieties with a tighter husk cover store better than some improved varieties. Husk cover was regarded as one of the variables that differentiates maize variety susceptibility to seed-borne fungal isolates. Bakan (2002) [23] reported the need to genetically modify maize for resistance to insect damage in order to reduce pathogenic fungal infection in stored maize grains. Moreover, attention should be paid to avoid pathogens in both certified and farmer-saved seed maize as these can be major sources of infection as well as continuous cropping [12].

The results of mycotoxin detection demonstrated that fungal isolates from the cereal crops studied produced secondary metabolites regarded as deleterious [2]. This is also indicated by the studies carried out by [4]. The mycotoxin types present and their amounts could be attributed to period, grain stored, and various management practices at the farm level. Sinha and Sinha [28] carried out a study that showed aflatoxin levels in relation to the storage period in stored maize and rice. The results from this study support the findings by Franzolin et al. [22] who showed that management practices play a role in the development of fungi and aflatoxins. The factors can be intercropping effects and laying maize plants on the soil and collecting the cobs later.

Orsi et al. [5, 29] reported similar mycotoxin-producing pathogens identified in this study. The mycoflora in postharvest and stored maize was analysed, and similar results were also found on mycotoxin-forming ability of seed-borne fungal pathogens by Pozzi et al. [30]. Aldred and Magan [31] showed that the incidence of *A. flavus* as well as the level of aflatoxins were comparatively more in maize samples having insect damage than the undamaged ones, which is in line with the findings of this research.

Aldred and Magan [31] found out that mycotoxins can be produced by fungi in maize prior to harvest, but their quantities generally increase after harvest, particularly during the storage period. Drying of maize is a preventative way for medium- and long-term storage in clean facilities, with no insect infestations and microorganisms. Moreover, regulation of grain moisture levels will significantly cause a reduction of mycotoxins in maize [17]. However, according to the Food and Agriculture Organisation [32] and Food and Drug Administration regulations, the mycotoxins that were detected in the samples tested in this study were within the safe limits. Therefore, the grain is rendered safe for human or animal consumption.

## 5. Conclusion and Recommendations

### 5.1. Conclusion

The fungal genera identified were *F*. *moniliforme*, *A*. *niger*, *A*. *flavus*, *A*. *tamarii*, *A. parasiticus*, *R*. *stolonifer*, and *P*. *citrinum*. Storage facilities as well as varietal types were the factors to be noted in the contribution of fungal contamination. Maize varieties showed differences in susceptibility to seed-borne fungal contamination. This study confirmed that storage facilities affect the type and level of fungal infection in stored grain. The results of this investigation show that some of the fungal species isolated from the seed have mycotoxigenic potential. Four types of mycotoxins, namely, zearelenone, aflatoxin total, deoxynivalenol (DON), and fumonisins, were detected in the maize samples. The evidence from this study thus suggests that knowledge of the fungal species and mycotoxins identified help develop effective control strategies.

## Figures and Tables

**Table 1:** Samples selected for mycotoxin concentration determination.

Sample number	Sample code	Storage facility/variety	Mycotoxin tested
1	F22	House sacks/PAN 513	DON
2	F27	House sacks/PAN 413	Aflatoxin total
3	F25	House sacks/PAN 513	DON
4	F9	Traditional granary/PIO 3253	Aflatoxin total
5	F23	Traditional granary/PIO 3253	Fumonisins
6	F24	House sacks/PIO 3253	Aflatoxin total
7	F1	House sacks/PIO 3253	Aflatoxin total
8	F40	Traditional granary/PIO 3253	Zearalenones
9	F93	Traditional granary/8 line	Zearalenones
10	F28	Traditional granary/PIO 3253/2859	Fumonisins

**Table 2:** Mean seed infections of fungal pathogens detected on maize grain stored in different storage environments.

Storage facility	*Fusarium moniliforme*	*Rhizopus stolonifer*	*Penicillium citrinum*
Kitchen	1.29^a^ (17.78)	0.67^a,b^ (8.89)	0.29^a,b^ (2.22)
Cotton bags	1.33^a,b^ (21.43)	0.86^b^ (6.67)	0.32^a,b^ (1.43)
Traditional granary	1.45^a,b^ (28.65)	0.76^a,b^ (4.76)	0.69^b^ (3.97)
House sacks	1.51^a,b^ (32.19)	0.68^a,b^ (3.88)	0.71^b^ (4.59)
Crib	1.52^b^ (33.81)	0.26^a,b^ (0.95)	0.26^a,b^ (0.95)
Container	1.90^c^ (80.0)	0.00^a^ (0.00)	0.00^a^ (0.00)

*p*-value	<0.001	0.035	0.021
LSD	0.172	0.544	0.412
CV (%)	6.3	55.6	46.7
SE	0.077	0.244	0.1849

Means followed by different letters in superscript in the column denote significant differences as determined by Tukey's test.

**Table 3:** Mean seed infections by *Aspergillus* species (fungal pathogens) detected on stored maize grain samples.

Storage facility	*Aspergillus flavus*	*Aspergillus parasiticus*	*Aspergillus niger*
Kitchen	0.42^a^ (2.22)	4.14^b^ (17.8)	1.36^a,b^ (2.22)
Cotton bags	0.75^a,b^ (6.19)	0.71^a^ (0.00)	3.35^c,d^ (0.95)
Traditional granary	1.18^b^ (14.29)	1.24^a^ (1.2)	4.25^d^ (17.62)
House sacks	1.13^b^ (12.46)	1.4^a^ (3.2)	3.70^c,d^ (13.22)
Crib	0.43^a,b^ (2.86)	1.98^a^ (4.3)	2.30^b,c^ (5.24)
Container	0.00^a^ (0.00)	0.71^a^ (0.00)	0.71^a^ (6.00)

*p*-value	0.006	0.002	<0.001
LSD	0.561	1.35	0.962
CV (%)	47.3	41.9	20.2
SE	0.252	0.606	0.432

Means followed by different letters in superscript in the column denote significant differences as determined by Tukey's test.

**Table 4:** Mycotoxin levels detected in 10 maize samples in ppb.

Sample number	Sample code	Mycotoxin tested	Amount (ppb)
1	F22	DON	2.30
2	F27	Aflatoxin total	0.80
3	F25	DON	3.00
4	F9	Aflatoxin total	0.08
5	F23	Fumonisins	0.08
6	F24	Aflatoxin total	0.00
7	F1	Aflatoxin total	0.14
8	F40	Zearalenones	0.80
9	F93	Zearalenones	4.05
10	F28	Fumonisins	0.06

## Data Availability

The data used to support the findings of this study are available from the corresponding author upon request.
